# An antidote approach to reduce risk and broaden utility of antibody-based therapeutics

**DOI:** 10.1074/jbc.M117.775528

**Published:** 2017-03-03

**Authors:** Alyse D. Portnoff, Cuihua Gao, M. Jack Borrok, Xizhe Gao, Changshou Gao, G. Jonah Rainey

**Affiliations:** From the Departments of ‡ Antibody Discovery and Protein Engineering and; § Translational Sciences, MedImmune, Gaithersburg, Maryland 20878

**Keywords:** antibody engineering, biotechnology, chemical biology, drug metabolism, Fc receptor, immunoglobulin G (IgG), monoclonal antibody, pharmacokinetics, receptor recycling

## Abstract

Antibody therapeutics offer effective treatment options for a broad range of diseases. One of the greatest benefits of antibody therapeutics is their extraordinarily long serum half-life, allowing infrequent dosing with long-lasting effects. A characteristic of antibodies that drives long half-life is the ability to interact with the recycling receptor, FcRn, in a pH-dependent manner. The benefit of long half-life, however, carries with it liabilities. Although the positive effects of antibody therapeutics are long-lasting, any acute adverse events or chronic negative impacts, such as immunosuppression in the face of an infection, are also long-lasting. Therefore, we sought to develop antibodies with a chemical handle that alone would enjoy the long half-life of normal antibodies but, upon addition of a small-molecule antidote, would interact with the chemical handle and inhibit the antibody recycling mechanism, thus leading to rapid degradation and shortened half-life *in vivo*. Here we present a proof of concept study where we identify sites to incorporate a non-natural amino acid that can be chemically modified to modulate FcRn interaction *in vitro* and antibody half-life *in vivo*. This is an important first step in developing safer therapeutics, and the next step will be development of technology that can perform the modifying chemistry *in vivo*.

## Introduction

Therapeutic antibodies continue to push the boundaries of disease treatment through specificity, efficacy, functionality, and potency (1, 2). Currently there are more than 400 antibody therapeutics in clinical development for diseases ranging from cancer to autoimmune disease to infectious disease (3). As the landscape of diseases to be treated and mechanisms of action diversify for antibodies, it will also be critical to mitigate risks associated with these potent molecules. For example, improved effector function engineering and broad immune effector modulation through immune checkpoint therapies could pose greater risks than existing treatments, albeit with potential greater benefit (1, 2, 4–6). Also, if risks can be mitigated, therapeutic options can be used more broadly and extended to non-life-threatening diseases, such as migraines and chronic pain (3, 7). Finally, there may be a desire to clear an antibody used for chronic dosing temporarily, such as immunosuppressive antibodies in the presence of infection (8).

**Figure 1.**
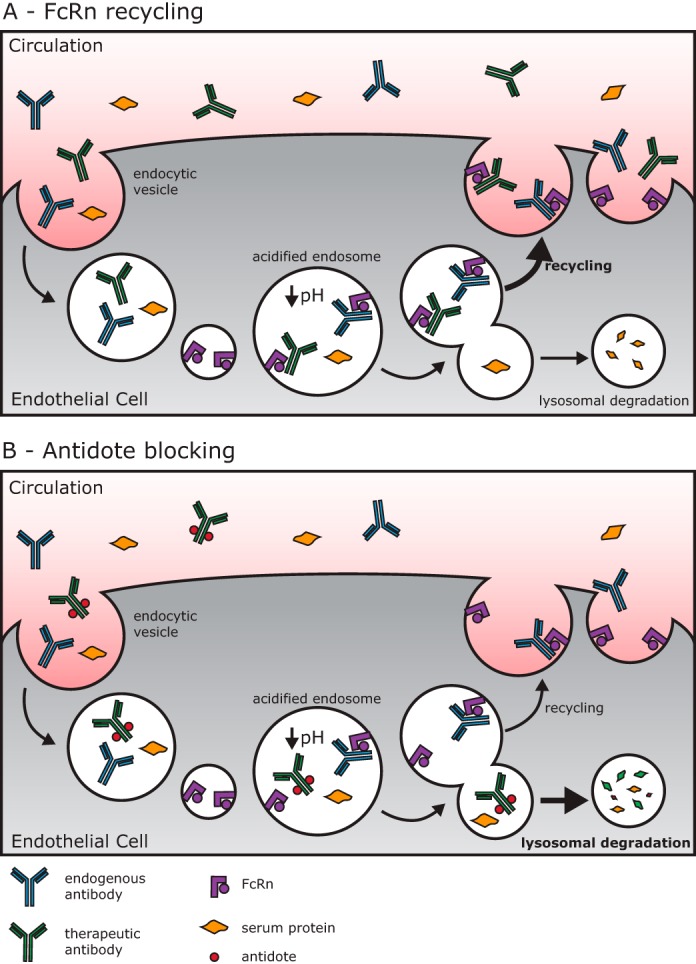
**Schematic of antidote mechanism.** *A*, the role of FcRn recycling for circulating antibodies. Proteins in circulation are taken up by fluid phase pinocytosis by endothelial cells where the majority of antibodies bind FcRn in the mildly acidic early endosome. Then proteins bound to FcRn are trafficked to a recycling endosome and returned to the cell surface where they dissociate and return to circulation at the nearly neutral pH of the serum. *B*, the antidote blocks FcRn binding, and the therapeutic antibody follows the default fluid phase endocytic path to the lysosome for degradation.

In recognition of the need to mitigate risks associated with therapeutic antibodies, we set out to develop an antidote specific for antibody therapeutics. Ideally this antidote would enable specific and fast removal of the therapeutic antibody without affecting endogenous antibody levels or the patient's immune system. Therapeutic antibodies have a naturally long half-life of ∼21 days because of their large size, allowing them to escape renal clearance, and their pH-dependent interaction with the neonatal Fc receptor, FcRn, which regulates IgG homeostasis (9–12). During circulation, serum proteins are regularly pinocytosed by vascular endothelial cells (13). FcRn, present in endosomes, is able to protect antibodies from lysosomal catabolism by binding them in the mildly acidic environment of the early endosome and releasing them into circulation at neutral pH after recycling to the cell surface (14–16). However, if the therapeutic antibody were modified by an antidote that blocked FcRn binding, then the antibody would proceed along the default path to lysosomal degradation (Fig. 1) (13, 17). In this work we propose to exploit the FcRn salvage mechanism to modulate antibody half-life with the goal of mitigating risks associated with therapeutic antibodies. By on-demand blocking of the FcRn binding site of the therapeutic antibody with an antidote, the FcRn recycling mechanism would be inhibited, and the half-life of the therapeutic antibody would be significantly reduced (Fig. 1). To achieve this goal, we have created a chemically addressable FcRn modulator.

Specifically, we have introduced a non-natural amino acid (nnAA)^3^ into the Fc domain of a therapeutic antibody (18, 19). This nnAA can be incorporated site specifically at an amber stop codon by using the orthogonal pyrrolysyl-tRNA synthetase (pylRS) and its cognate tRNA from *Methanosarcina mazei* (20, 21). By incorporating the aliphatic nnAA *N*^ϵ^-((2-azidoethoxy)carbonyl)-l-lysine (AzK) into the Fc domain, we introduced a bio-orthogonal chemical handle (20). This azide group can then be covalently modified by an antidote containing a strained alkyne through strain promoted azide-alkyne cycloaddition (SPAAC), one variety of “click” chemistry (18, 22, 23). In this study, we have identified sites in the Fc domain to accommodate the nnAA AzK incorporation while maintaining native FcRn affinity, thus retaining natural half-life. We also found the incorporated AzK to be amenable to chemical conjugation for modulating the FcRn affinity. Finally we evaluated proof-of-concept antidotes that can block FcRn binding, demonstrating increased antibody clearance *in vivo*.

## Results

### Identifying sites in the FcRn:Fc interface to test for nnAA incorporation

**Figure 2.**
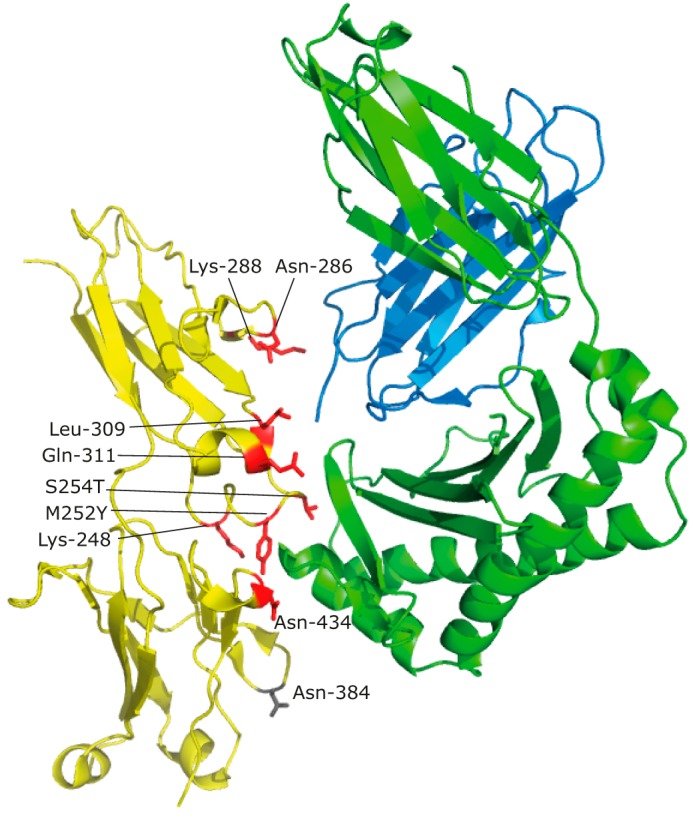
**Three-dimensional structure identifying sites in the Fc:FcRn interface for nnAA incorporation.** FcRn α-chain, β_2_-microglobulin, and Fc domains are shown in *green*, *blue*, and *yellow*, respectively. Sites selected for nnAA incorporation in the Fc:FcRn interface are highlighted in *red* with the distant control site, Asn-384, identified in *gray*. Protein Data Bank file 4NOU with Fc mutations M252Y, S254T, and T256E was used to create the model, and the figure was generated in PyMOL (DeLano Scientific).

To modulate FcRn binding, we evaluated the co-crystal structure of the antibody constant domain and human FcRn to identify amino acids at the binding interface that could accommodate a non-natural amino acid (Fig. 2) (24). Two native lysine residues, Lys-248 and Lys-288, which are near the interface but not directly involved in binding, were chosen for conservation of structure to the lysine derivative AzK. Additionally, Asn-286, which is near the β_2_-microglobulin interface but does not directly contribute to binding, was chosen to test. Amino acid mutations M252Y/S254T/T256E, T250Q/M428L, and N434A have previously been shown to improve FcRn affinity, suggesting that these sites are amenable to mutation (24–27). However, Thr-250 and Met-428 are more internal to the CH_2_-CH_3_ interface rather than oriented toward the FcRn interface and thus were not chosen for testing (24). Additional sites Leu-309 and Gln-311 interface with the α-chain and β_2_-microglobulin, contributing to hydrophobic interactions and a hydrogen bond, respectively. Thus a collection of eight different antibody mutants was cloned with the amber stop codon, UAG, at sites Lys-248, Met-252, Ser-254, Asn-286, Lys-288, Leu-309, Gln-311, and Asn-434 to test their capacity to accommodate AzK while maintaining FcRn binding (Fig. 2). Notably we have not altered the histidine residues (His-310, His-433, and His-435) in the FcRn binding site known to influence the pH-dependent capability of IgG to bind FcRn (24, 28, 29). An additional site, Asn-384, was chosen as a negative control site for incorporation of AzK distant from the FcRn binding site (24). Finally, all of these sites are solvent-accessible to enable efficient antidote conjugation (24).

### Determining sites in the Fc domain that can accommodate AzK incorporation and maintain FcRn binding

**Figure 3.**
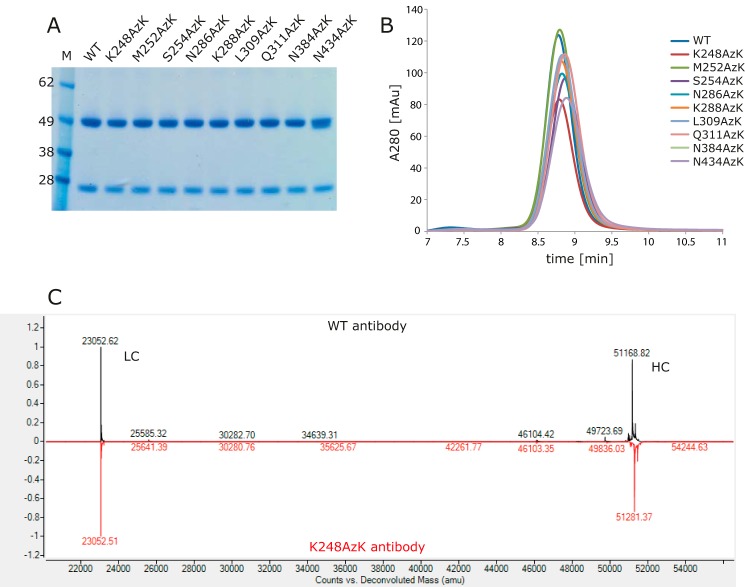
**Purified antibodies containing AzK are efficiently expressed and purified.** *A*, SDS-PAGE evaluation of purified antibodies under reducing conditions. *B*, overlay of size exclusion chromatography of intact antibodies. *C*, rLC/MS of reduced wild-type antibody (*black*) compared with the incorporation of AzK (113 Da) at amino acid site Lys-248 (*red*).

Incorporation of AzK was accomplished by co-transfection of the antibodies with an orthogonal pylRS/tRNA pair derived from *M. mazei* in CHO cells (20, 22). In this expression system, incorporation of the nnAA competes with amber stop codon termination, leading to truncated species; therefore purified antibodies were analyzed by mass spectrometry and SDS-PAGE to confirm the AzK incorporation and full-length species (Fig. 3 and supplemental Fig. S1). Incorporation of the nnAA AzK was successful for all evaluated sites, and only one site (Asn-434) showed truncated species remaining post-purification. This is likely because the protein A-binding site is downstream of most of the nnAA incorporation sites, and any truncate species therefore would not be capable of binding protein A (30, 31). SDS-PAGE demonstrated that only normal full-length HC and LC were detectable for all variants, except for Asn-434 (Fig. 3*A*). No aggregate or fragment species were detectable for WT or any of the mutants when they were analyzed by SEC (Fig. 3*B*). Any species truncated at Asn-434 would not be expected to exhibit a distinct SEC profile because they would only be a few amino acids shorter than WT. Finally, Fig. 3*C* shows mass spectrometry results of WT antibody compared with K248AzK. As expected, the LC mass is unaltered, and the HC mass is 113 Da larger than WT because of incorporation of the nnAA. Supplemental Fig. S1 (*A–H*) demonstrates that the AzK is efficiently incorporated into all sites, and any prematurely terminated species are not purified by protein A for these mutants. In contrast to the rest of the mutants, N434AzK shows a significant amount of truncated species for HC in the protein A-purified product (supplemental Fig. S1*I*).

A critical element of creating a therapeutic antibody with FcRn modulation is that the naturally long half-life of the antibody be maintained in the absence of the antidote. Thus the eight different sites of nnAA incorporation in the FcRn binding interface were evaluated for steady-state binding to human FcRn by Biacore (Table 1). Surprisingly, AzK incorporation in the Fc domain at sites known to directly interact with FcRn did not alter the *in vitro* binding affinity for FcRn and at a number of sites modestly improved the *in vitro* FcRn affinity (fold increase in KD from WT < 1). The control site, N384AzK, distant from the FcRn binding interface, did not show a significant change in FcRn affinity as expected.

**Table 1:** **Equilibrium affinity for huFcRn of antibodies containing AzK in the Fc domain** Steady-state affinity measurements were made using a Biacore 3000 with antibodies immobilized at 3000 RU and huFcRn flowed at 5 μl/min for 50 min at pH 5.8 over a concentration range from 19.5 nm to 10 μm. Equilibrium data were fit using BIAevaluation software. At least two experiments were performed for each construct, and reported data include duplicate concentrations utilized for data fitting.

Antibody	huFcRn pH 5.8 *K_D_*	Fold change from WT
	μ*m*	
WT	0.538	1.00
K248AzK	0.378	0.70
M252AzK	0.249	0.46
S254AzK	0.465	0.86
N286AzK	0.377	0.70
K288AzK	0.418	0.77
L309AzK	0.437	0.81
Q311AzK	0.323	0.60
N384AzK	0.565	1.05
N434AzK	0.734	1.36

### Evaluating SPAAC antidote candidates for conjugation and FcRn blocking

Having identified a collection of sites that maintain native huFcRn affinity, we next tested their ability to be conjugated with the strained alkyne dibenzocyclo-octyne (DBCO) (32). To evaluate the size and flexibility requirements of proof-of-concept antidotes to block FcRn binding, we tested four different molecules: DBCO-biotin; two fluorophore conjugates, DBCO-CR110 and DBCO-Cy5; and finally a linear PEG conjugate DBCO-mPEG (Table 2).

**Table 2:**
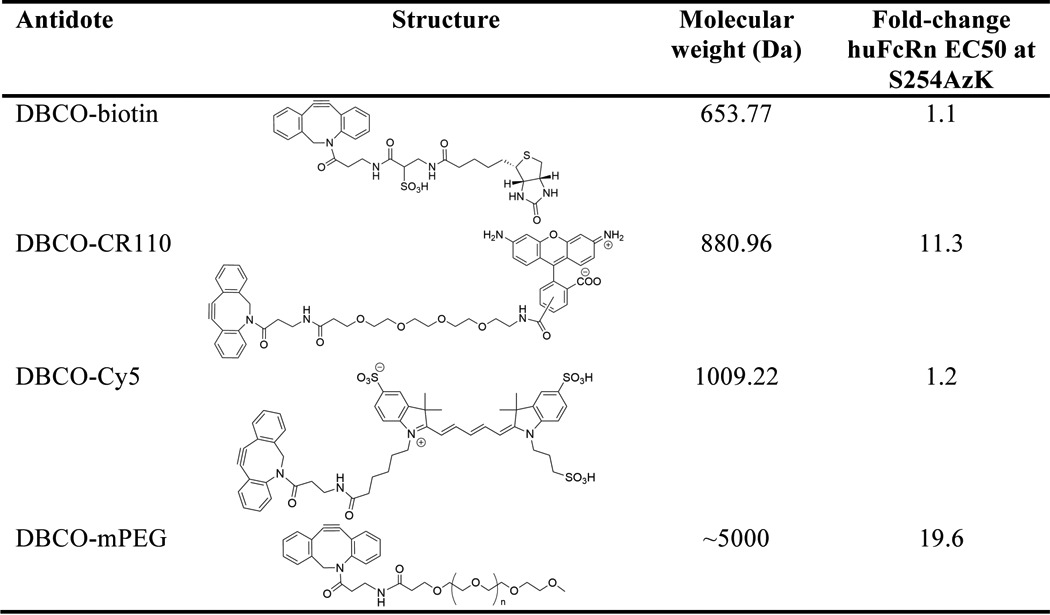
**Strained alkyne antidote molecules** All DBCO reagents were purchased from click chemistry tools.

**Figure 4.**
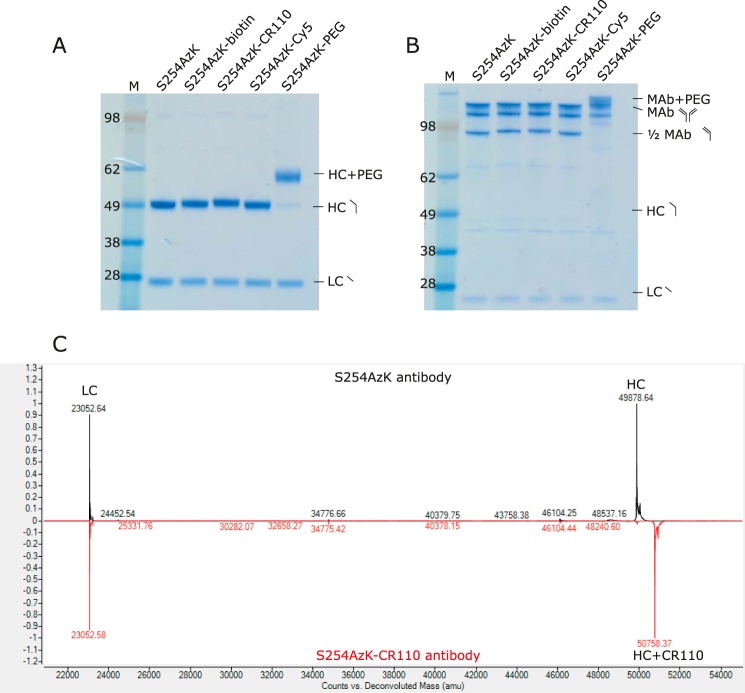
**Antibodies are efficiently conjugated with antidotes using SPAAC.** *A* and *B*, SDS-PAGE of SPAAC conjugation at site S254AzK with DBCO reagents evaluated under reducing (*A*) and non-reducing (*B*) conditions. *C*, rLC/MS of reduced S254AzK antibody (*black*) compared with the post-conjugation sample with DBCO-CR110 (*red*) showing the expected shift of 880 Da on the heavy chain.

SPAAC reactions at the S254AzK position showed nearly complete conjugation efficiency as evaluated by SDS-PAGE and mass spectrometry (Fig. 4 and supplemental Fig. S2). Under reducing conditions, all of the light chains migrate similarly to the unmodified S254AzK control. However, the heavy chains of the conjugated molecules all migrate more slowly than the unconjugated control, most strikingly for the S254AzK-PEG, which is the largest modification by mass (Fig. 4*A*). Similar results were obtained by non-reducing SDS-PAGE, with a small amount of half-antibody detectable for both the unconjugated control and the conjugated species (Fig. 4*B*). Because of the relatively low resolution of SDS-PAGE to decisively determine mass differences in this range, mass spectrometry analysis was also performed. This analysis confirmed the efficiency and the specificity of the chemical reactions. As can clearly be seen for the DBCO-CR110 conjugate, a reduced intact mass shift of 879.73 Da is observed (Fig. 4*C*), very similar to the 880.96 Da mass of the DBCO-CR110 used to conjugate (Table 2). Also clear from the mass spectrometry is that no detectable addition to the light chain takes place, no detectable unconjugated material remains, and there is no detectable signal of any species with multiple conjugations. Similar results were obtained for all of the other conjugated antidotes (supplemental Fig. S2). Next, the ability of each of the antidotes to block FcRn binding was evaluated by ELISA at the S254AzK position. This position was chosen as the test case because of its unaltered FcRn binding and significant interactions with the α-chain of FcRn (24). FcRn binding similar to the unconjugated control was observed for DBCO-biotin and DBCO-Cy5. By contrast, both DBCO-CR110 and DBCO-mPEG conjugates showed considerably reduced binding to FcRn of ∼10- and 20-fold, respectively (Table 2 and supplemental Fig. S3).

The two favorable antidote candidates, DBCO-CR110 and DBCO-mPEG, were next conjugated to all AzK sites previously examined in Fig. 3, except the N434AzK mutant because of the impurity of truncated species. Antibodies conjugated with the antidotes were then evaluated for FcRn affinity by Biacore at each of the AzK sites to identify the optimal site for FcRn binding modulation (Table 3). Site of conjugation clearly influenced the capacity for FcRn blocking as evidenced by the minimal change observed at sites Lys-248, Lys-288, and the distant site Asn-384. Of these sites, the Lys-248 side chain does not directly point toward the FcRn α-chain, and whereas the Lys-288 side chain does directly point toward the β_2_-microglobulin, only one residue (Thr-86) of β_2_-microglobulin is within 4 Å of Lys-288 (24). However, numerous sites showed reduced FcRn binding in the presence of the conjugated antidotes. The most dramatic reductions in FcRn binding were observed at sites Met-252, Leu-309, and Gln-311, each having close to 4-fold reduction in binding with DBCO-CR110 conjugated and up to 7-fold reduction in binding with DBCO-mPEG conjugated. Of these sites, Met-252 interacts dominantly with the α-chain of huFcRn, whereas Leu-309 and Gln-311 interface with both the α-chain and β_2_-microglobulin (Fig. 2) (24). Additionally, sites Ser-254 and Asn-286 both had reductions in FcRn binding with the conjugated antidotes when evaluated by Biacore. From the *in vitro* evaluations of antidote blocking at various sites in the Fc domain, it was evident that the longer, more flexible 5-kDa mPEG antidote generally outperformed the smaller fluorophore antidote candidate. One exception to this trend is S254AzK, which shows a more potent reduction in affinity with the smaller CR110 compared with mPEG and the most potent reduction of any site when conjugated with CR110.

**Table 3:** ***In vitro* evaluation of antidote blocking FcRn binding** Steady-state affinity measurements were made using a Biacore 3000 with antibodies immobilized at 3000 RU and huFcRn flowed at 5 μl/min for 50 min at pH 5.8 over a concentration range from 19.5 nm to 10 μm. Equilibrium data were fit using BIAevaluation software. Samples for which conjugation caused a greater than 3-fold reduction in affinity relative to WT are shown in bold type. At least two experiments were performed for each construct, and reported data include duplicate concentrations utilized for data fitting.

Antibody	huFcRn pH 5.8 *K_D_*	Fold change from WT
	μ*m*	
WT	0.538	1.00
K248AzK	0.378	0.70
K248AzK-CR110	0.489	0.91
K248AzK-mPEG	0.907	1.69
M252AzK	0.249	0.46
M252AzK-CR110	**2.06**	**3.83**
M252AzK-mPEG	**3.58**	**6.65**
S254AzK	0.465	0.86
S254AzK-CR110	**2.87**	**5.33**
S254AzK-mPEG	1.54	2.86
N286AzK	0.377	0.70
N286AzK-CR110	1.10	2.04
N286AzK-mPEG	**2.83**	**5.26**
K288AzK	0.418	0.78
K288AzK-CR110	0.558	1.03
K288AzK-mPEG	1.12	2.08
L309AzK	0.437	0.81
L309AzK-CR110	**2.04**	**3.79**
L309AzK-mPEG	**3.27**	**6.08**
Q311AzK	0.323	0.60
Q311AzK-CR110	**2.05**	**3.81**
Q311AzK-mPEG	**3.87**	**7.19**
N384AzK	0.565	1.05
N384AzK-CR110	0.702	1.30
N384AzK-mPEG	0.756	1.41

### In vivo clearance of antibodies containing AzK and preconjugated with antidote

**Figure 5.**
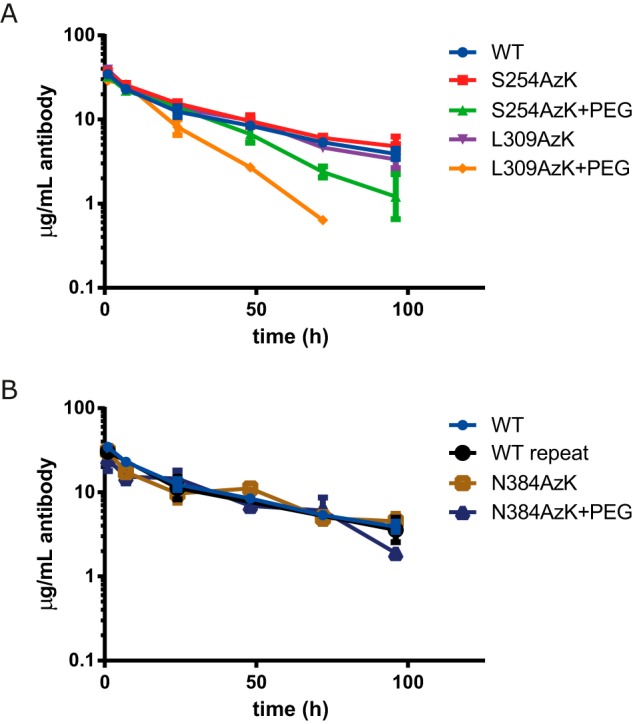
**Antidotes that block FcRn binding profoundly impact antibody PK in huFcRn transgenic mice.** *A*, antibody serum concentration curves for wild-type (*blue*), S254AzK (*red*), S254AzK-PEG (*green*), L309AzK (*purple*), and L309AzK-PEG (*orange*) in huFcRn transgenic mice. The final 96 h data point for L309AzK-PEG was below the limit of detection. *B*, N384AzK (*brown*) control and PEG-conjugated antibody (*navy*) are shown compared with WT.

Although we envision the ultimate application of this technology to be implemented by administering antibody and then administering antidote on demand *in vivo*, we first wanted to conduct a proof of concept study in which we measured the pharmacokinetic (PK) properties of antibodies modified prior to administration compared with WT and AzK mutants that had not been modified as controls. Based on the loss of FcRn binding of the DBCO-mPEG-conjugated L309AzK and Q311AzK demonstrated by Biacore (Table 3), these sites were considered for PK testing in a huFcRn transgenic mouse model. Ultimately, L309AzK was selected over Q311AzK because unconjugated Q311AzK had an almost 2-fold improvement in FcRn binding, potentially confounding analysis. Additionally the more subtle affinity modulation found with S254AzK modified with DBCO-mPEG was evaluated to compare sites and to determine the impact of different levels of FcRn affinity reduction *in vivo*. Thus the wild-type antibody, L309AzK, L309AzK conjugated with DBCO-mPEG (L309AzK-PEG), S254AzK, and S254AzK conjugated with DBCO-mPEG (S254AzK-PEG) were dosed in huFcRn transgenic mice to determine their half-lives and rates of serum clearance (Fig. 5*A*) (28). Additionally the control site N384AzK and N384AzK conjugated with DBCO-mPEG (N384AzK-PEG) were tested to confirm that increased clearance observed *in vivo* was due to blocking the binding of FcRn (Fig. 5*B*), not some artifact caused by PEG conjugation. Table 4 summarizes the relevant pharmacokinetic parameters determined by fitting the serum clearance data.

**Table 4:** **Pharmacokinetic parameters for antibodies tested in huFcRn transgenic mice**

Antibody	*C*_max_	AUC_INF_	*C*_L_	*t*_1/2_	*V*_ss_
	μ*g/ml*	*day*·μ*g/ml*	*ml/day/kg*	*Day*	*ml/kg*
WT	34.58	52.36	38.20	1.77	87.78
S254AzK	36.58	60.93	32.82	1.74	76.46
S254AzK-PEG	32.69	38.21	52.34	0.81	62.16
L309AzK	38.93	52.15	38.35	1.40	70.84
L309AzK-PEG	28.85	27.21	73.50	0.54	51.39
N384AzK	30.88	52.34	38.21	1.96	105.75
N384AzK-PEG	24.61	40.83	48.98	1.28	85.22

As predicted by the *in vitro* binding analysis, incorporation of AzK alone at sites Ser-254, Leu-309, and Asn-384 had no significant impact on serum clearance or antibody half-life. These values are typical for human antibodies in the human FcRn transgenic mouse and are reflected in the WT control and recapitulated elsewhere (28). Further, N384AzK-PEG had PK properties similar to WT and unconjugated controls, with only slightly increased clearance compared with WT antibody and unconjugated control (Fig. 5*B* and Table 4), indicating that PEGylation itself does not significantly impact the PK properties of antibodies in this experimental system. By contrast, L309AzK-PEG showed markedly faster clearance, whereas S254AzK-PEG had a more moderate half-life reduction, both of which proportionally reflect the reduced FcRn affinities determined *in vitro*. Thus this study clearly demonstrates the ability to use blocking of FcRn binding to modulate the half-life of a therapeutic antibody *in vivo*.

## Discussion

Developing an antidote for antibody based therapeutics will provide a valuable safety switch built into therapeutic molecules with expanding applications and increasing potency. Previous efforts to negatively modulate FcRn binding by antibodies have focused on increasing the overall clearance by saturating the neonatal Fc receptor (29). Although this approach could be used to clear therapeutic antibodies, it would also clear endogenous antibodies, and maintenance of the patient's immune repertoire may be critical in the face of disease. In this work we have developed a proof-of-concept approach for removing a therapeutic antibody from circulation on demand while leaving the endogenous antibodies intact. Using a structure-based approach informed by the co-crystal structure of the human IgG1 Fc domain with FcRn, we were able to modulate the half-life of a therapeutic antibody with the site-specific conjugation of a non-natural amino acid.

This study represents an important first step in proving that this is a viable approach to selectively modulate serum levels of therapeutic antibodies *in vivo*. However, progress toward finding more efficient bio-orthogonal *in vivo* conjugation chemistries needs to be made. We have found that the SPAAC reaction between the strained alkyne DBCO and an azide incorporated tag suffers from slow reactivity and low efficiency in serum, presumably because of the rate of reaction (data not shown). This result is in agreement with previously reported evaluation of *in vivo* click chemistry limitations (33). For our antidote approach to be realized *in vivo*, it will require highly stable reactants with strict specificity, faster reactivity, and nearly complete efficiency. Solutions may include alternative click chemistry options such as the inverse Diels-Alders reaction using a tetrazine and *trans*-cyclooctene (34, 35) or potentially very high affinity non-covalent interactions (36). However, each of these technologies will need to be examined in the context of non-natural amino acid incorporation into the therapeutic antibody and reaction specificity in serum. In this work, we have also only evaluated a small collection of possible antidote molecules (Table 2). We were able to achieve up to 7-fold reduction in FcRn affinity, resulting in a doubling of clearance rate and a 2.6-fold reduction in terminal serum half-life. However, it is possible that further optimization in blocking the FcRn binding could be achieved with larger antidote molecules such as branched PEG (37). Optimization of the antidote could enable faster clearance and thus develop a stronger safety switch.

Selective clearance technology has a large number of important applications. Many therapeutic antibodies used for control of inflammation also cause immunosuppression (8). This technology would allow the patient to selectively remove immunosuppressive therapeutics in the face of an acute infection and then to resume therapeutic antibody treatment once the infection was cleared. Further, many antibody therapeutics are safe for the majority of patients but may lead to adverse events in a subset that does not have clearly predictive measurable markers. This technology would allow patients to receive beneficial medicines while facilitating safe removal of the therapeutic in the face of severe adverse events. Finally, a great strength of antibody therapeutics is the infrequent dosing because of their intrinsically long half-life in serum. Indeed, several technologies have been developed to further extend half-life up to 60 days in humans, allowing for very infrequent dosing (25, 38, 39). One limitation to this extended half-life technology arises from concerns the possibility that potential adverse events could not be easily mitigated because of the extended washout period. Selective clearance technology will allow broader implementation of half-life extension technologies by providing a safety switch in the event that extremely long-lived therapeutics lead to unacceptable adverse events.

Here we have reported a proof of concept study identifying antibody Fc residues close to the FcRn binding site that can be modified to modulate FcRn binding and antibody clearance *in vivo*. This work represents an important first step toward developing therapeutic antibodies with safety switches to enable benefit to broader patient populations.

## Experimental procedures

### Construction, expression, and purification of antibody variants

Antibody positions are numbered using the Eu numbering system (40). The wild-type antibody was cloned into a proprietary mammalian expression vector encoding the light and heavy chains under control of the human cytomegalovirus promoter. The amber stop codon (TAG) was cloned into Fc positions using overlap extension PCR and InFusion® HD cloning (Takara 638911). Plasmids encoding the orthogonal pylRS and cognate tRNA for non-natural amino acid incorporation, pCEP4-pylRS and pOriP-9x-tRNA, respectively, were previously described (22). CHO cells (proprietary strain) were transiently co-transfected with the plasmid DNA of the antibody, pylRS, and tRNA at a 30:20:50 ratio using PEI max. For non-natural amino acid incorporation, AzK (SynChem SC-36462) was added to the medium 6 h post-transfection at the final concentration of 2 mm. Cultures were fed with a proprietary nutrient mixture, and conditioned medium was harvested 13 days post-transfection. Antibodies were purified using HiTrap protein A HP columns (GE Healthcare, catalog no. 17-0403-01) and dialyzed into PBS at 4 °C for 24 h. Purified antibodies were analyzed by SDS-PAGE, reduced reverse phase liquid chromatography (rLC)/MS, and HPLC-SEC to confirm full-length species and monomer content (Fig. 3 and supplemental Fig. S1).

### Expression and purification of huFcRn

Recombinant human FcRn was cloned into a proprietary mammalian expression vector encoding both the human α (FCGRT) and β (β_2_ microglobulin)-chains. A second expression vector was made with the C terminus of the α-chain appended with a dual AviTag® and His_6_ tag for *in vivo* biotinylation and purification purposes, respectively. HEK 293 cells (proprietary strain) were transfected using 293fectin (Invitrogen 12347019) and standard protocols. Cells were fed with both Gibco® FreeStyle^TM^ 293 expression medium (Invitrogen catalog no. 12338018) and a proprietary in-house feed. For *in vivo* biotinylation, huFcRn was co-transfected with BirA, and media were supplemented with 100 μm biotin, 10 μm ATP, and 100 μm Mg^2+^. Conditioned medium was harvested 10 days post-transfection and adjusted to pH 5.8 for purification over IgG-Sepharose 6 Fast Flow resin (GE Healthcare catalog no. 17-0969-01) as previously described (24). Purified huFcRn was dialyzed into PBS, pH 5.5, at 4 °C for 24 h and analyzed by SDS-PAGE and HPLC-SEC to confirm purity.

### Analytical methods

HPLC-SEC was used to evaluate the monomeric content of purified proteins. This was performed on a TSK gel G3000SW_XL_ column (Tosoh Bioscience catalog no. 08541) with a mobile phase of 0.1 m Na_2_SO_4_ and 0.05% NaN_3_ in 0.1 m sodium phosphate buffer at pH 6.8 at a flow rate of 1 ml/min. SDS-PAGE analyses were performed using precast NuPAGE® Novex® 4–12% Bis-Tris gels (Invitrogen NP0321) run with NuPAGE® MOPS SDS running buffer (Invitrogen catalog no. NP001) under standard conditions. Protein samples were prepared with NuPAGE® LDS sample buffer and reducing agent (Invitrogen catalog nos. NP0007 and NP0004) to evaluate protein purity and size and run with SeeBlue® Plus2 prestained protein standard (Invitrogen catalog no. LC5925).

rLC/MS was used to confirm the incorporation of the nnAA and conjugation of antidote candidates. This was performed with an Agilent 1200 series HPLC coupled to an Agilent 6520 accurate mass Q-TOF LC/MS with an electrospray ionization source as previously described (41). Proteins were first reduced with 50 mm DTT at 37 °C for 15 min at 0.5 μg/ml, and then 2 μg of reduced antibody was loaded onto a Poroshell 300SB-C3 column (2.1 × 75 mm, Agilent catalog no. 660750-909) and eluted at a flow rate of 0.4 ml/min using a step gradient of acetonitrile with 0.1% formic acid into water with 0.1% formic acid (JT Baker catalog nos. 9832-3 and 9834-23) after 6 min. Agilent MassHunter software was used for data acquisition and processing.

### huFcRn ELISA

Nunc® Immobilizer^TM^ streptavidin 96-well white plates (VWR catalog no. 73521-414) were prewashed with PBST at pH 6 prior to coating with 0.25 μg/ml of AviTagged huFcRn for 1 h. All incubations and washes were performed at room temperature in PBST at pH 6. Washed plates were next blocked with 5% blocking reagent (Bio-Rad catalog no. 1706404) for 2 h. This was followed by the introduction of serial antibody dilutions in 2.5% blocking reagent for 1 h. After washes to remove non-bound proteins, AffiniPure goat anti-human IgG1 (H+L) HRP antibody (Jackson ImmunoResearch catalog no. 109-035-088) was added at a 1:10,000 dilution in 2.5% blocking reagent for 1 h. For detection of the bound antibodies, the washed plates were incubated with Pierce Supersignal ELISA Pico Chemiluminescent substrate (Thermo Fisher catalog no. 37069) for 5 min at room temperature followed by chemiluminescent detection using an EnVision plate reader (PerkinElmer Life Sciences). The data were analyzed in Prism (GraphPad) using the non-linear fit, sigmoidal dose response to determine EC_50_ values.

### Surface plasmon resonance measurements

The binding affinity of huFcRn (produced at MedImmune) to purified antibodies was determined using a Biacore 3000 (GE Healthcare) as previously described (42). Briefly, antibodies were immobilized to a CM5 sensor chip (GE Healthcare catalog no. 29149604) using amine coupling at a surface density of ∼3000 RU. Purified huFcRn in pH 5.8 PBS containing 5 mm EDTA and 0.05% Tween 20 was then flowed over the surface at 5 μl/min for 50 min to reach steady-state. huFcRn concentrations ranged from 10 μm to 19.5 nm. The surface was regenerated using two 2-min pulses of PBS, pH 7.4, containing 0.05% Tween 20. Dissociation constants were determined by fitting the steady-state data using BIAevaluation Software. At least two experiments were performed for each construct, and reported data include duplicate concentrations utilized for data fitting.

### SPAAC conjugation of antidotes

DBCO reagents, DBCO-CR110, DBCO-Cy5, and DBCO-Biotin were reconstituted in DMSO at 1 mg/ml, whereas DBCO-mPEG 5 kDa was reconstituted at 5 mg/ml (Click Chemistry Tools catalog nos. A118-25, A127-25, A130-1, and A116-10). For SPAAC conjugations, antibodies at a final concentration of 4 mg/ml were incubated with 6-fold molar excess of DBCO reagents in PBS, pH 7.2, containing 10% DMSO at room temperature for 20–24 h (22). Unreacted DBCO reagents were removed using an illustra NAP-5 Sephadex G-25 column (GE Healthcare catalog no. 17-0853-02) pre-equilibrated with PBS. Conjugation efficiency was evaluated using rLC/MS for all antidotes, and DBCO-mPEG conjugation was also evaluated by SDS-PAGE (Fig. 4).

### In vivo pharmacokinetics in huFcRn transgenic mice

Pharmacokinetic analysis of antibodies was performed as previously described (28). Differences include using six mice per antibody, with two groups of three mice each bled at alternate time points. All mice were dosed with 2.0 mg/kg of antibody on day 0. For the quantitative ELISAs, serum samples were diluted 1:400 for earlier time points and 1:50 for late time points. Standard curves were generated for each antibody variant diluted in the same serum concentration as the samples using mouse serum obtained 3 days prior to dosing. The linear portions of standard curves were then used to quantify the IgGs in the serum samples, and the concentration of antibody remaining over time was graphed in Prism (GraphPad).

## Author contributions

A. D. P., M. J. B., Ch. G., and G. J. R. conceived of the hypothesis and designed the research activities. A. D. P. and Cu. G. performed the experiments and analyzed the data. X. G. interpreted the results and performed PK modeling. A. D. P. wrote the manuscript. Ch. G. and G. J. R. provided critical review and edited the manuscript.

## Supplementary Material

Supplemental Data

## Footnotes

3 The abbreviations used are:

nnAA: non-natural amino acid
pylRS: pyrrolysyl-tRNA synthetase
AzK: *N*^ϵ^-((2-azidoethoxy)carbonyl)-l-lysine
SPAAC: strain promoted azide-alkyne cycloaddition
DBCO: dibenzocyclo-octyne
PK: pharmacokinetic
rLC: reduced reverse phase liquid chromatography
SEC: size exclusion chromatography
RU: response unit(s).
