# Supplementary material for: An antidote approach to reduce risk and broaden utility of antibody-based therapeutics
Source: J Biol Chem. 2017 Mar 3;292(20):8498–506. doi: 10.1074/jbc.M117.775528 (PMC5437253; doi:10.1074/jbc.M117.775528)
Supplement: Supplemental Data [file supp_292_20_8498__index.html]

An antidote approach to reduce risk and broaden utility of antibody based therapeutics — An antidote approach to reduce risk and broaden utility of antibody-based therapeutics — Antibody antidote — Supplemental Data 

# An antidote approach to reduce risk and broaden utility of antibody-based therapeutics

## Supplemental Data

- Supplemental data (.pdf, 654 KB) - Contains supplemental data to be published online along with article
