# Supplementary material for: An antidote approach to reduce risk and broaden utility of antibody-based therapeutics
Source: J Biol Chem. 2017 Mar 3;292(20):8498–506. doi: 10.1074/jbc.M117.775528 (PMC5437253; doi:10.1074/jbc.M117.775528)
Supplement: Supplemental Data [file 10.1074_M117.775528_jbc.M117.775528-1.pdf]

An Antidote Approach to Reduce Risk and Broaden Utility of Antibody Based Therapeutics

Alyse D. Portnoff, Cuihua Gao, M. Jack Borrok, Xizhe Gao, Changshou Gao, and G. Jonah Rainey

CONTENTS
3 Supplemental figures

Figure S-1

A

WT antibody

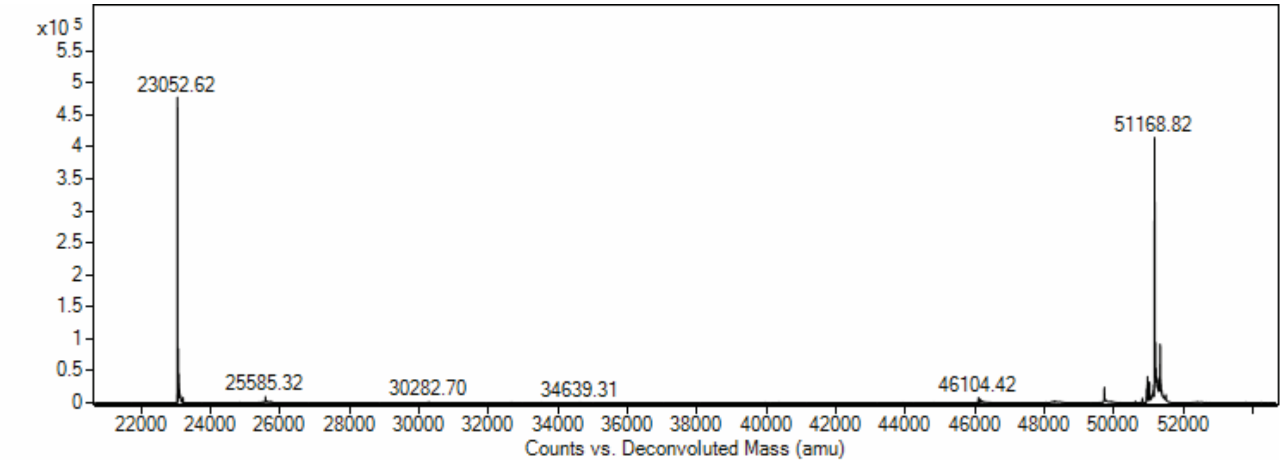

B K248AzK antibody

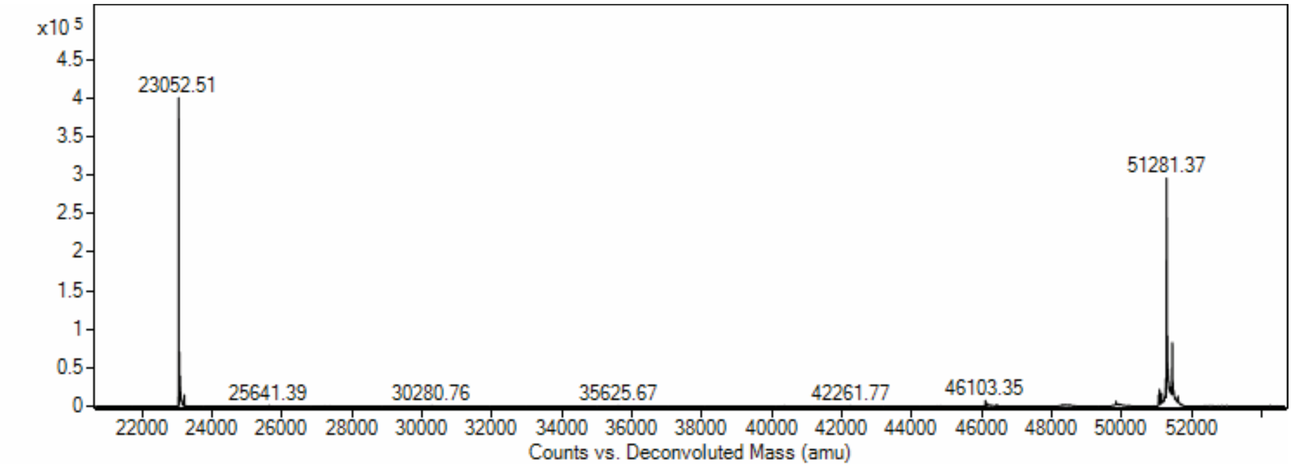

C

M252AzK antibody

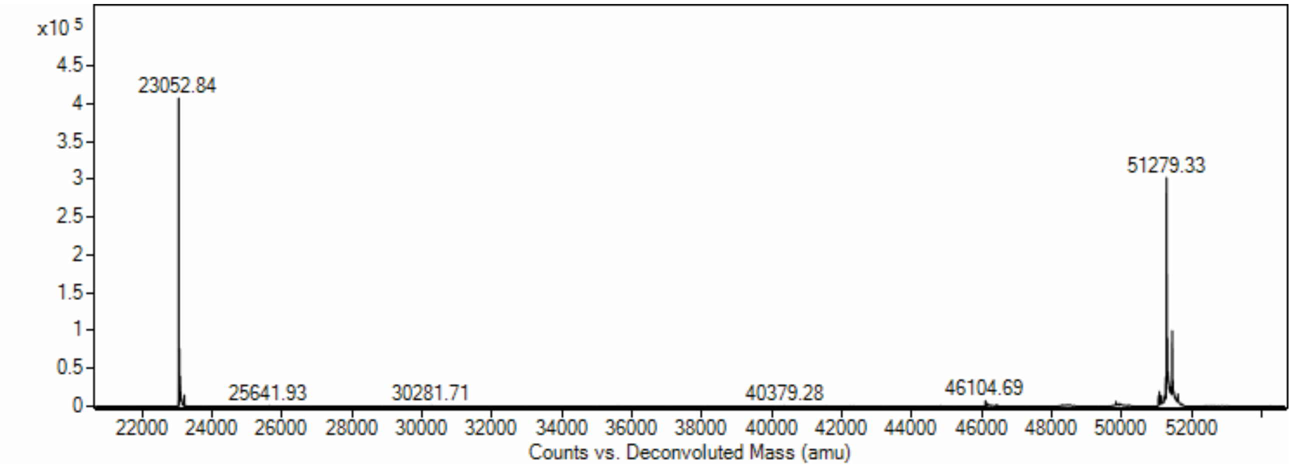

D

S254AzK antibody

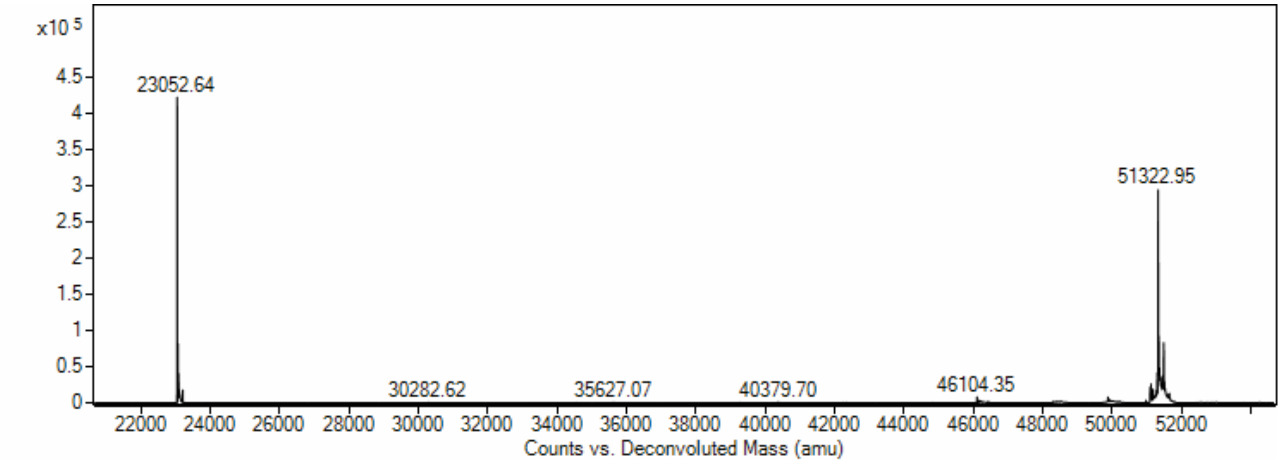

E N286AzK antibody

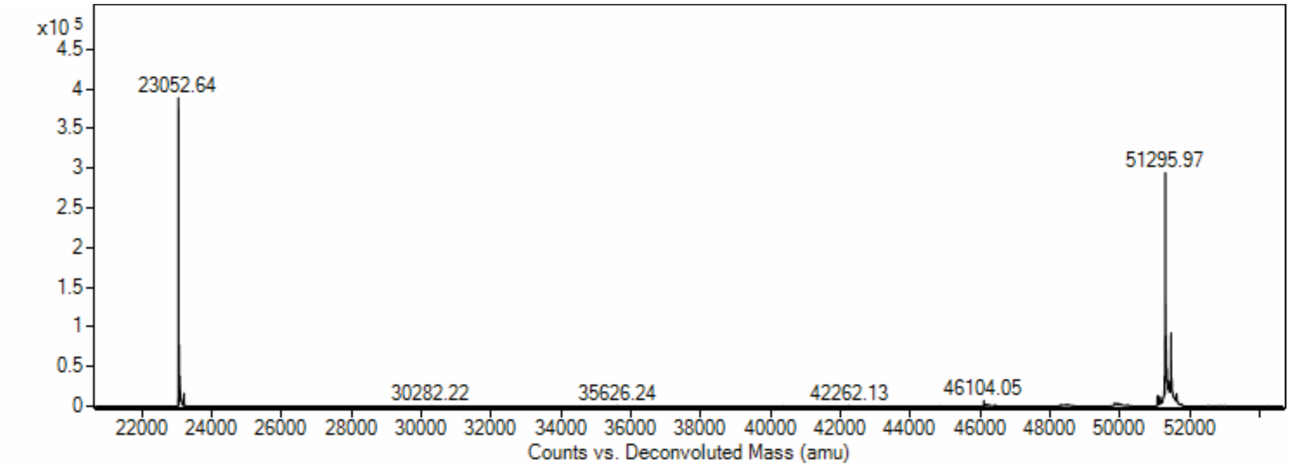

F K288AzK antibody

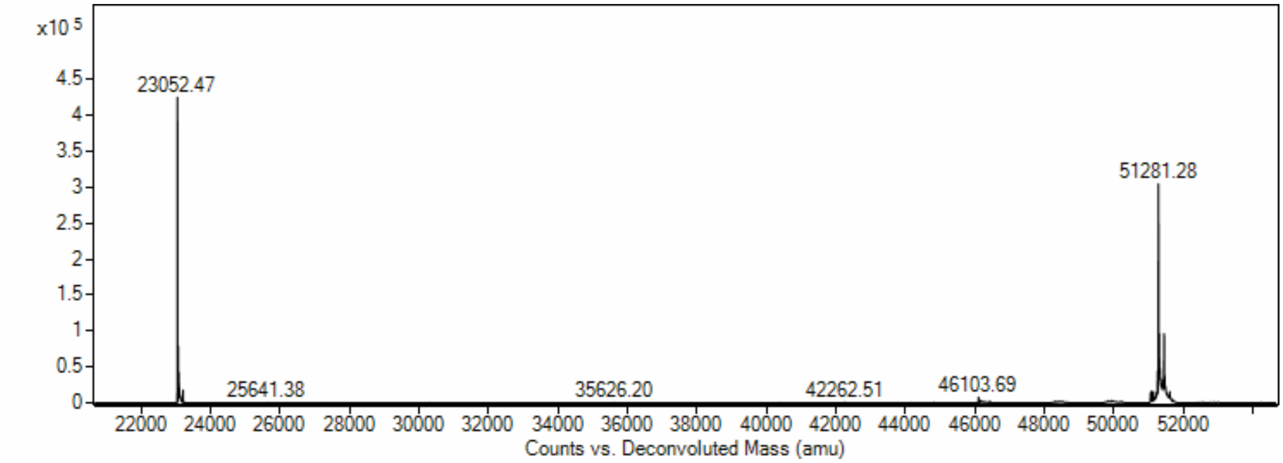

G

L309AzK antibody

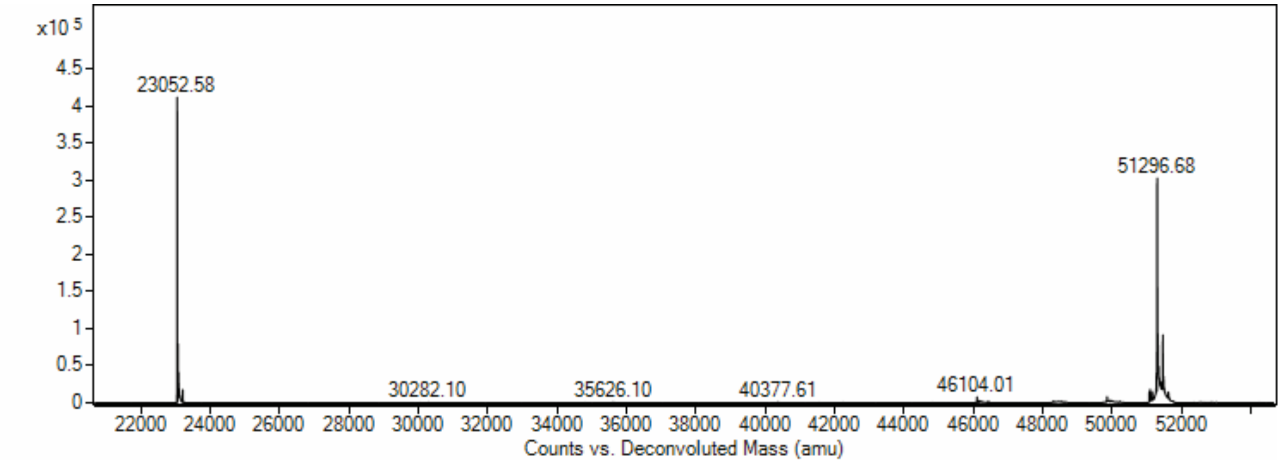

H Q311AzK antibody

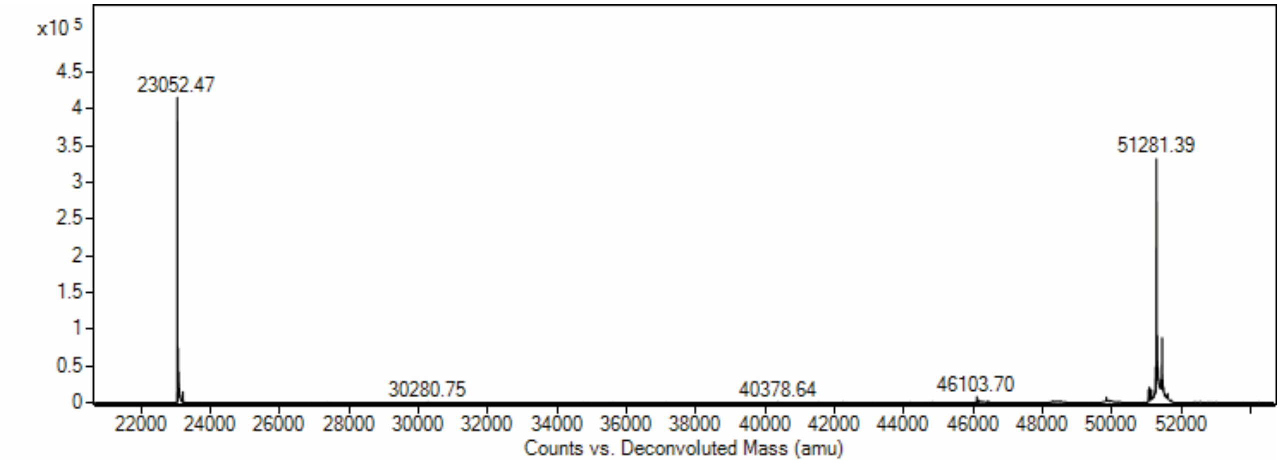

I N434AzK antibody

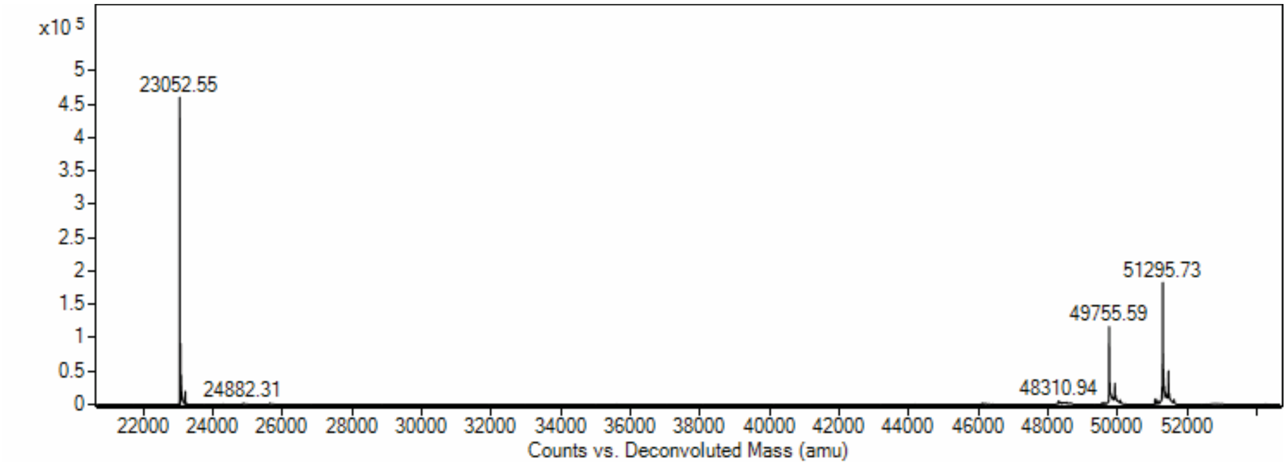

Figure S1. LC-MS of purified antibodies verifies efficient AzK incorporation

rLCMS of reduced purified antibodies demonstrating the incorporation of AzK at each amino acid site. Heavy-chain expected masses (Da): wild-type 51169, K248AzK 51282, M252AzK 51279, S254AzK 51323, N286AzK 51296, K288AzK 51282, L309AzK 51297, Q311AzK 51281, N434AzK 51296.

Figure S-2

A LC

S254AzK antibody unmodified

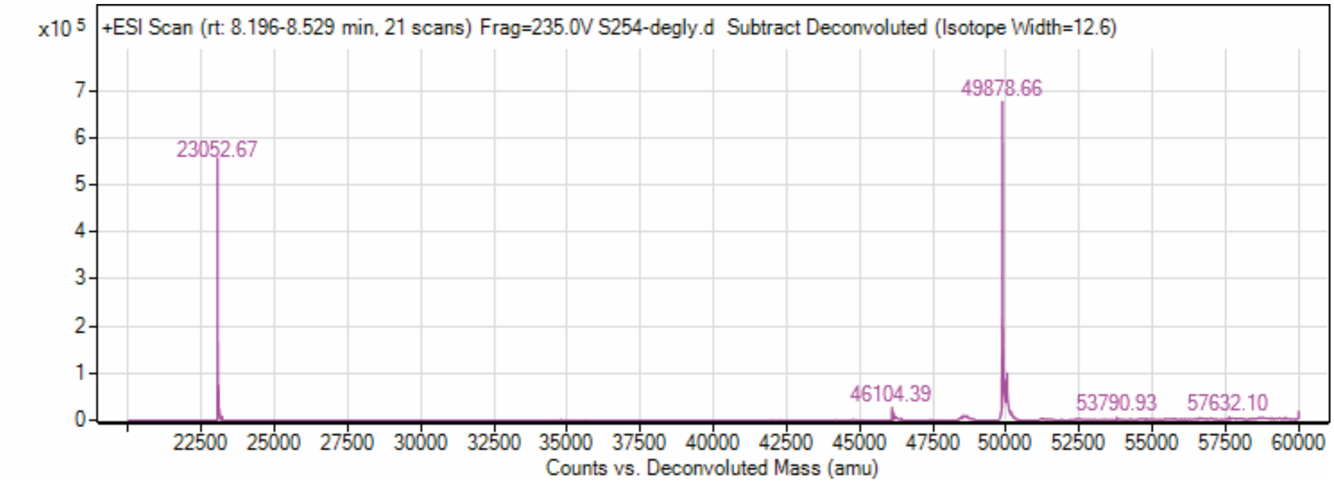

B S254AzK-Cy5 antibody LC

HC+Cy5

HC

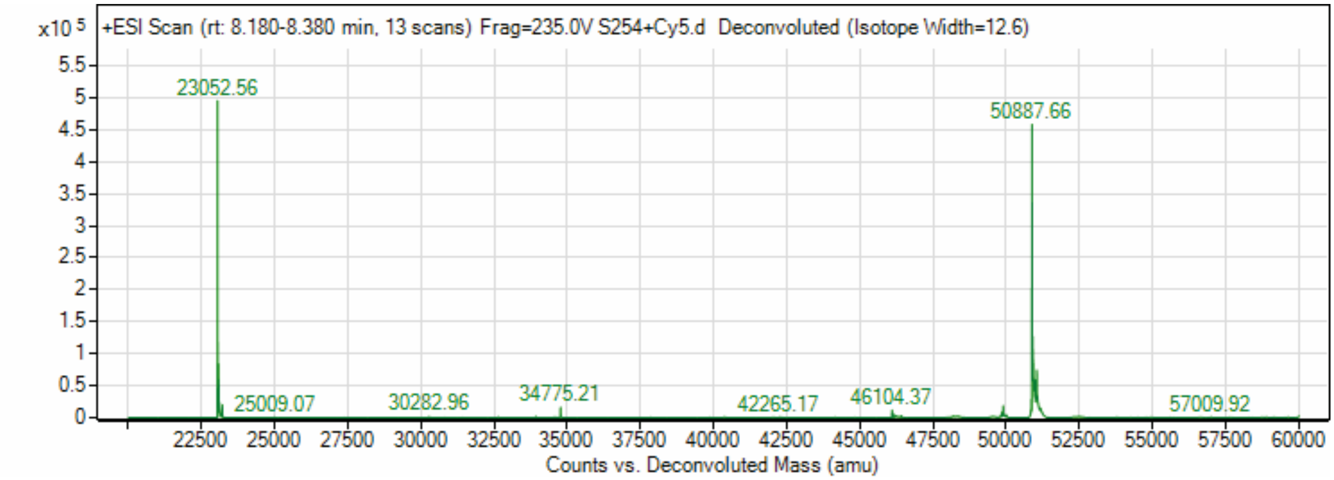

C LC

S254AzK-biotin antibody

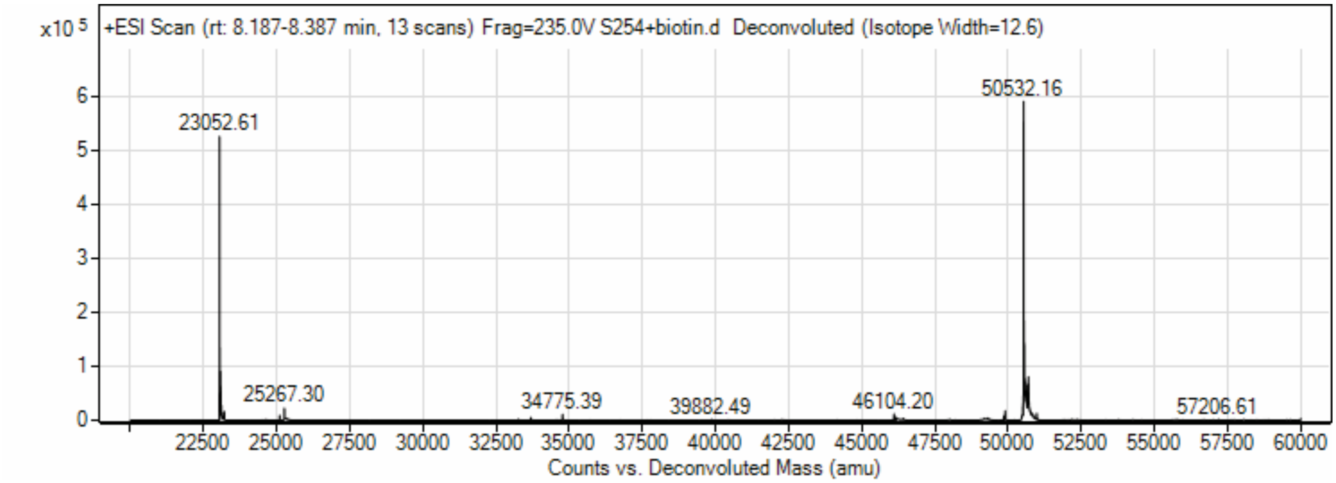

HC+biotin

D S254AzK-PEG antibody LC

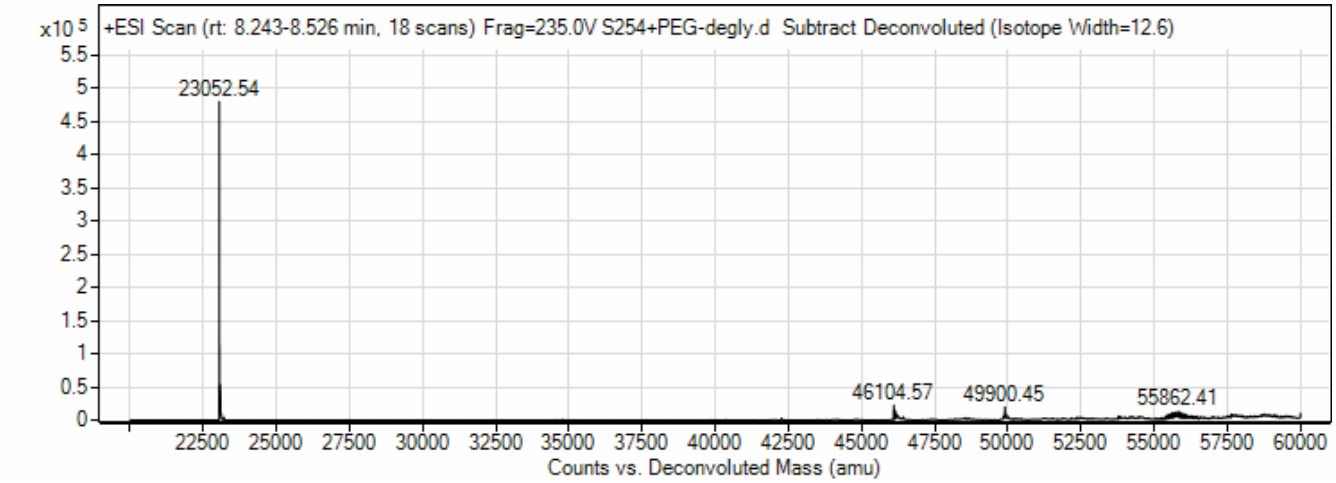

E S254AzK-PEG modified HC

HC+PEG

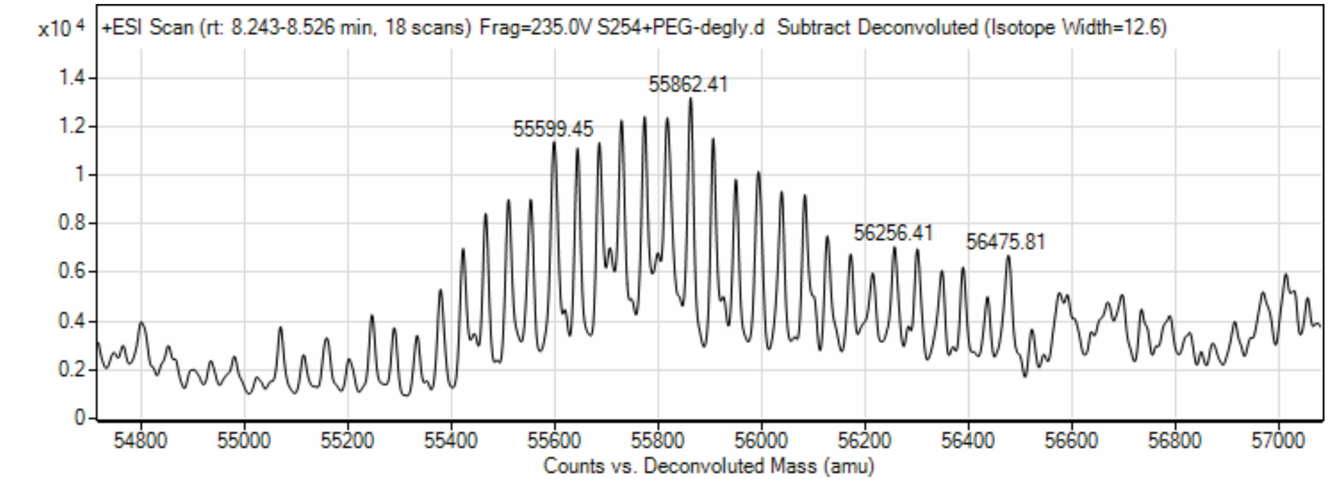

Figure S2. Antibody conjugation to a panel of antidotes

LC/MS results are shown indicating completeness of reaction and addition of mass consistent with conjugated antidotes. The S254AzK antibody before conjugation (A) and post-SPAAC conjugation with DBCO-Cy5 (B), DBCO-biotin (C), and DBCO-mPEG (D&E).

Figure S-3

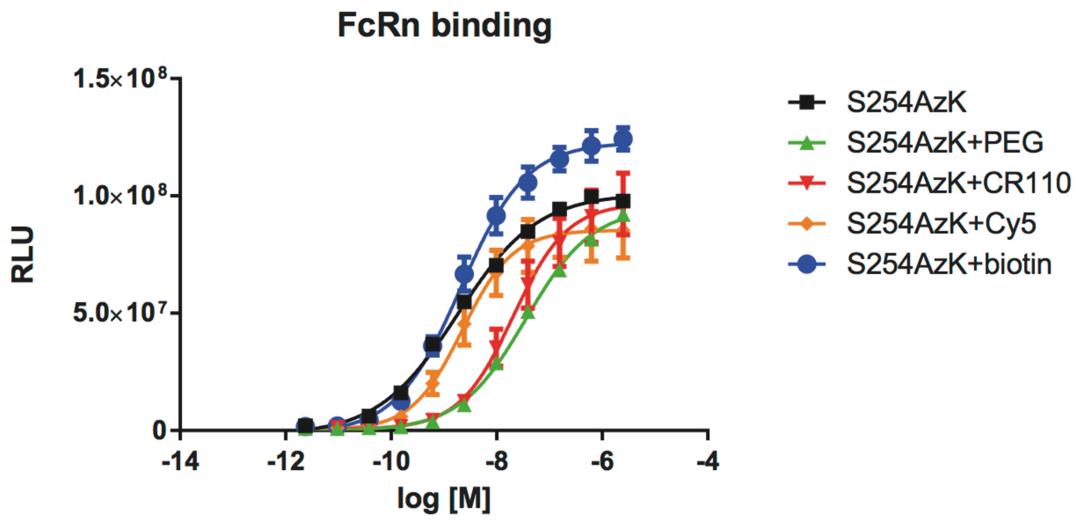

Figure S3. huFcRn ELISA evaluation of antidotes at S254AzK position

S254AzK conjugated with each of the four potential antidotes was bound to ELISA plates coated with huFcRn.
